# The Homeostatic Interaction Between Anodal Transcranial Direct Current Stimulation and Motor Learning in Humans is Related to GABA_A_ Activity

**DOI:** 10.1016/j.brs.2015.04.010

**Published:** 2015

**Authors:** Ugwechi Amadi, Claire Allman, Heidi Johansen-Berg, Charlotte J. Stagg

**Affiliations:** a Oxford Centre for Functional MRI of the Brain (FMRIB), Nuffield Department of Clinical Neurosciences, University of Oxford, John Radcliffe Hospital, Oxford OX3 9DU, UK; b Oxford Centre for Human Brain Activity (OHBA), Department of Psychiatry, University of Oxford, Warneford Hospital, Oxford OX3 7JX, UK

**Keywords:** Non-invasive brain stimulation (NIBS), Motor learning, GABA, Homeostatic plasticity

## Abstract

**Background:**

The relative timing of plasticity-induction protocols is known to be crucial. For example, anodal transcranial direct current stimulation (tDCS), which increases cortical excitability and typically enhances plasticity, can impair performance if it is applied before a motor learning task. Such timing-dependent effects have been ascribed to homeostatic plasticity, but the specific synaptic site of this interaction remains unknown.

**Objective:**

We wished to investigate the synaptic substrate, and in particular the role of inhibitory signaling, underpinning the behavioral effects of anodal tDCS in homeostatic interactions between anodal tDCS and motor learning.

**Methods:**

We used transcranial magnetic stimulation (TMS) to investigate cortical excitability and inhibitory signaling following tDCS and motor learning. Each subject participated in four experimental sessions and data were analyzed using repeated measures ANOVAs and post-hoc *t*-tests as appropriate.

**Results:**

As predicted, we found that anodal tDCS prior to the motor task decreased learning rates. This worsening of learning after tDCS was accompanied by a correlated increase in GABA_A_ activity, as measured by TMS-assessed short interval intra-cortical inhibition (SICI).

**Conclusion:**

This provides the first direct demonstration in humans that inhibitory synapses are the likely site for the interaction between anodal tDCS and motor learning, and further, that homeostatic plasticity at GABA_A_ synapses has behavioral relevance in humans.

## Highlights

• We investigated the homeostatic relationship between motor learning and anodal tDCS.
• Anodal tDCS applied to M1 slowed learning of a subsequent motor task.
• The worsening of learning after tDCS was accompanied by an GABA_A_ activity increase.
• The learning worsening and GABA_A_ activity increase after tDCS were closely related.

## Introduction

Transcranial direct current stimulation (tDCS) is a non-invasive technique known to modulate cortical excitability [1]. Anodal tDCS, which increases cortical excitability, is attracting increasing interest as a putative adjunct therapy in the recovery of function after stroke [2], [3], [4], [5]. However, a number of questions remain as to *how* tDCS should be applied to optimize its behavioral effects, with particular reference to the relative timing of the stimulation and motor task.

In healthy controls, anodal tDCS has been shown to improve motor learning [6], [7], [8], but only when tDCS is applied *during* the learning task [6], [9]. When tDCS is applied prior to the task, the positive behavioral effects of tDCS are not seen [10], or even reversed [6].

These time-dependent interactions between tDCS and motor learning can provide insights into the mechanisms of action of tDCS applied to the motor cortex. Motor learning is dependent on Hebbian synaptic plasticity mechanisms such as Long-Term Potentiation (LTP)-like effects within the interneurons of the primary motor cortex [11], [12], [13]. LTP-like plasticity operates by positive feedback and therefore carries the potential to destabilize established cortical networks, leading to unregulated cortical activity and preventing further dynamic modulations [14]. In order to maintain neural activity within a useful dynamic range, regulatory homeostatic mechanisms have been proposed to operate, whereby a stimulus that characteristically *increases* cortical excitability can lead to *decreased* cortical excitability if applied after another excitatory stimulus [15].

Homeostatic interactions have been demonstrated in humans between tDCS and motor learning as described above [6], between tDCS and other non-invasive brain stimulation (NIBS) techniques [16], [17], [18], and between other NIBS techniques and motor learning [19]. Evidence from animal studies suggests that this homeostatic plasticity is synapse-specific [20] and therefore the effects are seen most clearly when both stimuli directly modulate activity across the same synaptic circuits.

One critical and as yet unanswered question is what neuronal processes subserve the previously observed homeostatic interaction between tDCS and motor learning. Previous studies have suggested that both anodal tDCS [21], [22] and motor learning [23], [24] separately modulate GABA_A_ synapses, leading to the tentative hypothesis that these GABAergic synapses may act as the site of homeostatic interactions between the two interventions when they are applied serially. However, no study to date has directly examined mechanisms underlying homeostatic interactions between tDCS and motor learning.

Here, we investigated the interaction between anodal tDCS and motor learning by testing the effects of online and offline anodal tDCS on motor learning performance, and on the accompanying changes in motor cortical excitability and inhibition.

Motor evoked potentials (MEPs) were used to measure motor cortical excitability. Short-interval intra-cortical inhibition (SICI) measurements with an interstimulus interval of 1 ms (1 ms SICI) and 2.5 ms (2.5 ms SICI) were acquired to differentiate between inhibitory effects due to extrasynaptic GABA_A_ tone, as indicated by changes in 1 ms SICI, or modifications in synaptic GABA_A_ activity, as indicated by changes in 2.5 ms SICI [25].

## Materials and methods

### Ethical approval

All protocols contained in this study were submitted to, and approved by, the East London REC1 (10/H0703/50). Written informed consent was obtained from all subjects and all experiments conformed to the standards set by the latest revision of the Declaration of Helsinki.

### Experimental overview

**Figure 1:**
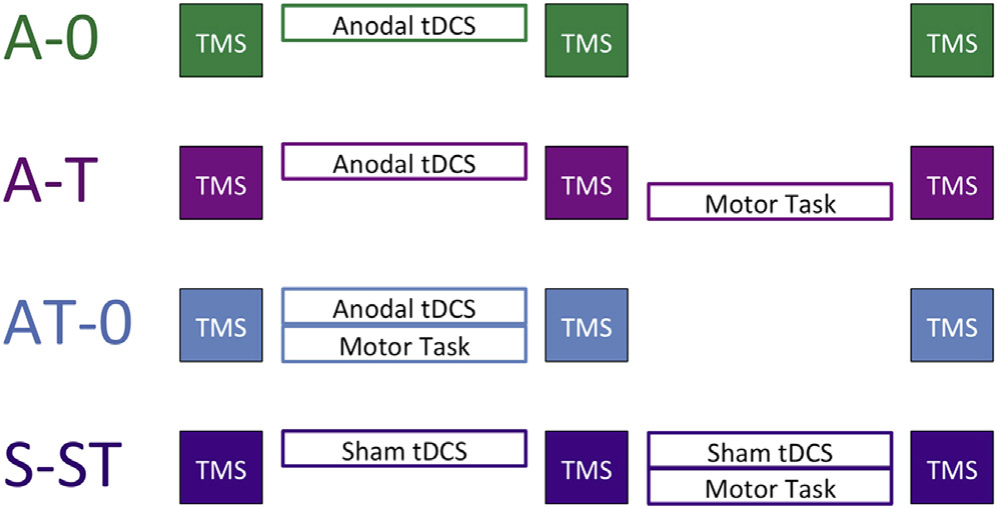
Schematic of experimental design. All subjects participated in all four experimental sessions, the order of which was counterbalanced across the group and all sessions were separated by at least 1 week. TMS blocks, consisting of 30 single pulses at MT_1mV_, 15 paired pulses with a 1 ms ISI and 15 paired pulses with a 2.5 ms ISI were acquired at the beginning, mid-point and end of each session. In the 20 min between TMS blocks, subjects had either anodal tDCS (A), sham tDCS (S) or no stimulation, while performing the motor learning task (T) or sitting quietly with their right hands relaxed.

Thirteen healthy, right-handed volunteers (6 male, mean age 24.5 y [range 23–29]) participated in each of the four sessions of this study, the order of which was counterbalanced across subjects and each session was separated by at least one week (Fig. 1). Briefly, each session consisted of periods in which TMS was used to assess cortical excitability and SICI, before and after modulation periods during which anodal (A) or sham (S) tDCS, a motor learning task (T), or rest (0), were delivered separately or in combination. Throughout the manuscript, the modulation period in question is highlighted in bold type (e.g., for the A-T session, the notation A-**T** indicates that the period for the motor learning task following a period of anodal tDCS is under consideration, whereas **A**-T indicates that the anodal tDCS period is under consideration). The two modulation periods were separated by approximately 5 min, during which TMS measures were acquired.

### TMS setup

TMS was applied using a 70 cm figure-of-eight coil attached to a BiStim module connecting two monophasic Magstim200 systems. The coil was held tangentially to the scalp and rotated 45° away from the midline. First, the ‘motor hotspot’ was localized, the optimal scalp position at which TMS evoked a just-noticeable twitch from the relaxed contralateral FDI muscle. Next, the TMS threshold required to evoke peak-to-peak MEP amplitudes of ∼1 mV on EMG from the contralateral FDI in 10/10 trials was identified (MT_1mV_). The active motor threshold (AMT) was determined during an isometric contraction of approximately 20% of maximum, and was defined as the lowest intensity necessary to evoke a 200 μV MEP on five out of ten trials.

### Motor-evoked potentials (MEPs)

Electromyographic (EMG) activity was recorded from the first dorsal interosseous muscle (FDI) of the participant's right hand in a belly-tendon montage using neonatal ECG electrodes (Tyco Healthcare, Germany). Participants were asked to relax their hand muscles during the experiment and compliance was monitored based on the background EMG. Responses were sampled, amplified and filtered using a CED 1902 amplifier, a CED micro1401 Mk.II A/D converter, and a PC running Signal (Cambridge Electronic Design, version 3.07). Signals were sampled at 5 kHz and band-pass filtered between 10 Hz and 1 kHz.

### Single and paired pulse measures

MT_1mV_ was identified at the beginning of the experiment. TMS measures were acquired in three blocks in each experimental session. At the beginning of each TMS block 10 single TMS pulses were applied at an intensity equal to MT_1mV_. In the Mid and Final TMS blocks of each session, if the MT_1mV_ had changed since the previous TMS block as evidenced by acquiring MEPs substantially (approximately 10%) larger or smaller than 1 mV, the stimulus intensity was adjusted until the elicited MEPs were again 1 mV in amplitude and this new MT_1mV_ was then used for the duration of the TMS block [22].

Paired-pulse TMS paradigms were used to elicit short interval intra-cortical inhibition (SICI) using interstimulus intervals (ISI) of 1 ms or 2.5 ms [25], [26], [27]. For the SICI measurements, the conditioning pulse was set to 70% of AMT, and the test pulse was set to MT_1mV_. During each TMS administration a total of 60 TMS pulses were delivered: 30 single pulses, 15 paired pulses with a 1 ms ISI and 15 paired pulses with a 2.5 ms ISI. The order of the conditions were applied in a pseudo-random order as generated within Signal.

### MEP data analysis

Analysis was conducted on the MEPs from each block separately. For each TMS pulse EMG activity in the 50 ms period prior to stimulation was analyzed, and any trace with visible pre-contraction of the FDI was excluded. Peak-to-peak amplitudes of the MEP from the 1 ms SICI, 2.5 ms SICI and single pulse trials were then calculated separately within Signal. A single iteration of Grubbs' test with a significance level of 0.05 was performed for each block of TMS and each trial type separately and significant outliers were excluded. Then, any MEPs that fell outside of two standard deviations from the mean were excluded from the analysis. The mean MEP amplitude was then computed for each trial type. For each paired pulse trial, the ratio of the conditioned MEP amplitude to the mean MEP amplitude from the single (unconditioned) pulses was calculated, and the mean of these ratios taken. Repeated measures ANOVAs, followed by *post hoc t*-tests for specific comparisons were performed in SPSS (IBM, version 21.0.0).

### Motor learning task

Three of the experimental sessions (A-T, AT-0 and S-ST) included performance of a visually-cued explicit sequence learning task lasting approximately 20 min. As described previously [28], four horizontal bars were displayed on the screen, each of which corresponded to a button on the button-box. The participants were told that when a bar changed into an asterisk, they were to press the corresponding button as quickly and accurately as possible. The task included sequence blocks consisting of three repeats of a ten-digit sequence. The first and fifteenth blocks consisted of 30 visual cues presented in a random order. Four sequences of equal difficulty were used in an order counterbalanced across the group; each sequence was constrained to the same ratio of button presses (3:3:2:2), to control for reaction time differences between fingers. Task blocks were of 30 s duration, and were separated by rest periods of 30 s.

Reaction time (RT) was calculated as the time from cue onset to a correct button press. Anticipatory responses, i.e. those that occurred prior to the cue, were discarded. RTs falling more than ±2 standard deviations from the mean were also excluded. Change in reaction times (ΔRT) was defined as the ratio of the mean reaction time for that block to the mean reaction time for the first learning block (block 2). The ΔRT for all of the sequence blocks for the task were then fed into a 3 by 15 repeated measures ANOVA including factors of stimulation condition (A-**T**, **AT**-0, and S-**ST**) and time (block number). In addition, to relate learning to changes in SICI, we needed to calculate a single metric representing learning. A learning measure was derived by calculating the mean ΔRT over blocks 10–14, when the learning plateaued [28].

### tDCS

Two electrodes measuring 5 × 7 cm were inserted in saline-soaked sheaths and placed on the scalp. The stimulating electrode was centered over the hand area measured as 5 cm lateral to Cz, as described previously [28], and the reference electrode was positioned over the contralateral supraorbital ridge. During real stimulation, 1 mA anodal tDCS was applied for 20 min via a DC-stimulator (Magstim Eldith) with a ramp up/ramp down of 10 s. For the sham condition, the current was ramped up for 10 s and then turned off.

## Results

### Baseline values

There were no systematic differences between the sessions in terms of the baseline measurements for MEP amplitude [RM-ANOVA main effect of session [*F*(3,36) = 2.102, *P* = 0.117]; MT_1mV_ [*F*(3,36) = 0.63, *P* = 0.443]; AMT [*F*(3,36) = 0.55, *P* = 0.650]; 1 ms SICI [*F*(3,36) = 1.719, *P* = 0.181]; and 2.5 ms SICI [*F*(3,36) = 0.377, *P* = 0.770]. There was also no difference between the average RT in the first block of the learning task across the three learning sessions [*F*(2,24) = 1.975, *P* = 0.161].

### Differential effects of online and offline tDCS on motor learning

**Figure 2:**
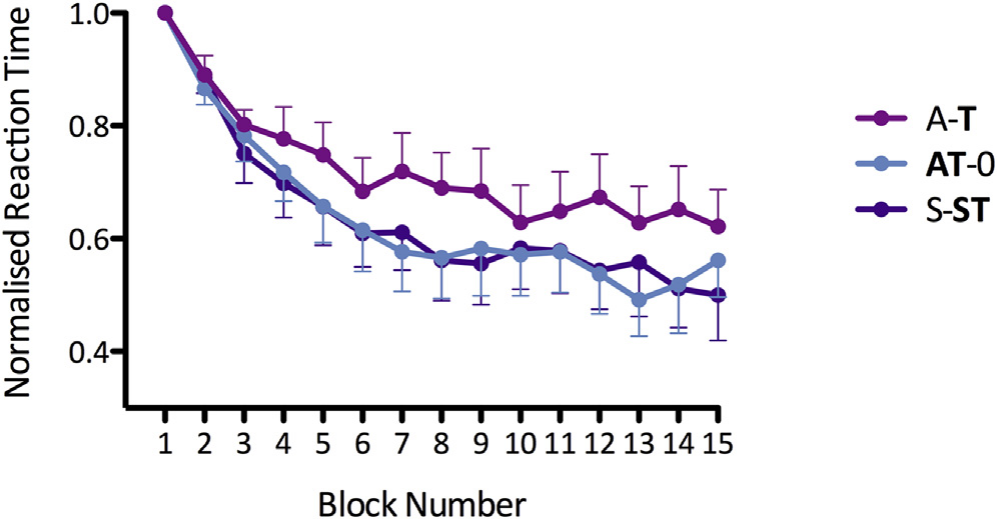
Motor learning task. Reaction times during the learning blocks of the motor task. Offline tDCS (A-**T**) led to decreased learning compared to online tDCS (**AT**-0) and control (S-**ST**). The two blocks containing random sequences (before Block 1 and after Block 13) have been excluded. Error bars ±1 SEM.

Reaction times decreased over time during the learning blocks (RM-ANOVA; main effect of time: *F*(14,168) = 19.278, *P* < 0.001; Fig. 2). To confirm that this decrease was due to learning of the sequences, we compared the reaction times in block 15 (the second random block) with the average reaction times from blocks 10–14 (representing the plateau of the learning). There was a significant difference in reaction times between the second random block and the learning plateau, suggesting that the decrease in reaction times represented learning of the sequence (Mean block RT in block 15: 353.3 ± 15.8 ms; mean RT in blocks 10–14: 226.2 ± 28.4 ms; RM-ANOVA main effect of block: *F*(1,12) = 51.155, *P* < 0.001; main effect of session: *F*(2,24) = 1.441, *P* = 0.256; session by block interaction: *F*(2,24) = 0.315, *P* = 0.733).

However, looking at the learning blocks alone, the degree of learning was not equivalent for the three sessions (RM-ANOVA, main effect of session: *F*(2,24) = 4.383, *P* = 0.024). Follow up ANOVAs between each pair of sessions established that this effect was driven by decreased learning when learning was preceded by tDCS (A-**T**) compared to other sessions (RM-ANOVAs main effect of session: A-**T***vs* S-**ST**: *F*(1,12) = 4.900, *P* = 0.047; A-**T***vs***AT**-0: *F*(1,12) = 4.877, *P* = 0.047; **AT**-0 *vs* S-**ST**: *F*(1,12) = 0.004, *P* = 0.949).

### Cortical excitability is modulated by tDCS and by task performance

To understand the physiological changes underpinning these behavioral effects, we first investigated the cortical excitability changes induced by anodal tDCS. As expected [1], [29], there was a significant increase in MEP amplitude after anodal tDCS compared with sham (RM ANOVA with one factor of session [**A**-0, **A**-T and **S**-ST] and one factor of time [pre, post] showed a session × time interaction *F*(2,24) = 6.89, *P* = 0.04). A follow-up RM ANOVA with one factor of session [**A**-0 and **A**-T] and one factor of time [pre post] demonstrated a main effect of time (*F*(1,12) = 6.59, *P* = 0.02), no main effect of session (*F*(1,12) = 0.318, *P* = 0.58) and no session × time interaction (*F*(1,12) = 4.07, *P* = 0.07). *Post hoc analyses* demonstrated an increase in MEPs with anodal tDCS [mean change due to stimulation in **A**-0 and **A**-T; paired *t*-test (pre-v post-stimulation); *t*(12) = 2.57, *P* = 0.02].

**Figure 3:**
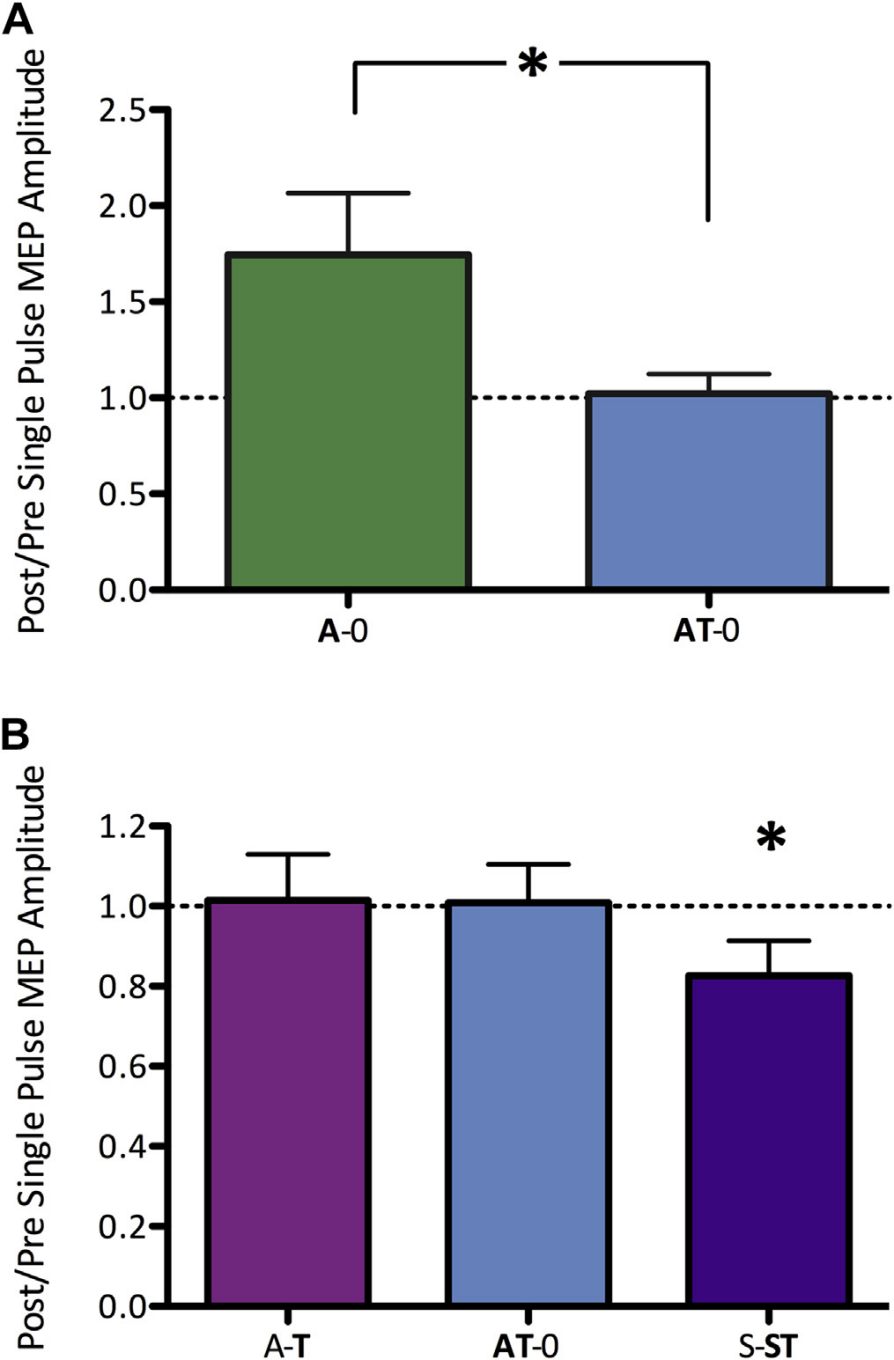
Effects of tDCS and Motor Learning on cortical excitability A. Anodal tDCS increases cortical excitability. Change in cortical excitability, expressed as a ratio of post-stimulation to pre-stimulation MEP size, where higher numbers reflect greater excitability. Anodal tDCS at rest (A-0) increases MEP size, an effect that is not seen if stimulation is applied concurrently with task performance (AT-0). * *P* < 0.05 B. Task performance decreases cortical excitability. Change in excitability, expressed as a ratio of post- to pre- task performance MEP size, where higher numbers reflect greater excitability. Performance of the task alone (S-ST) decreases MEP size, an effect that is not present if anodal tDCS is applied prior to (A-T) or concurrent with (AT-0) task performance. Error bars ±1 SEM, *P < 0.05.

We then investigated whether combining tDCS with learning had any significant effect on the increase in excitability seen with stimulation (i.e. comparing **A**-0 with **AT**-0). There was a significant difference between these two sessions in the change in MEP size due to stimulation (**A**-0 compared with **AT**-0, RM-ANOVA, session × time interaction: *F*(1,12) = 5.307, *P* = 0.040). Post-hoc tests confirmed that there was no increase in excitability when anodal tDCS was delivered during task performance (**AT**-0; paired *t*-test; t(12) = 0.077, *P* = 0.940; Fig. 3A).

We then asked what effect task performance had on cortical excitability. An RM-ANOVA comparing MEP size before and after task performance across the three task sessions (**AT**-0, A-**T** and S-**ST**) demonstrated a trend toward a change in MEP size with task (RM-ANOVA, main effect of time: *F*(1,12) = 4.124, *P* = 0.065). This task-induced trend toward a change in MEP size was not modulated by prior or concurrent tDCS (RM-ANOVA, time × session interaction: *F*(2,24) = 3.793, *P* = 0.313).

### Cortical inhibition: 1 ms and 2.5 ms SICI

We then went on to look at the effects of stimulation and task performance on measures of GABA signaling. First, to ensure that the magnitude of the test stimulus (TS) was consistent across time, we tested whether there was any difference between the MEP amplitude induced by the unconditioned TS in any of the TMS blocks. An RM-ANOVA demonstrated no main effect of time [Baseline, Mid, Final] across the sessions (*F*(2,24) = 0.9, *P* = 0.405) and no time × stimulation interaction (*F*(6,72) = 1.45, *P* = 0.208).

As expected, both the 1 ms SICI and 2.5 ms SICI protocols produced significant inhibition at baseline (i.e. the mean of conditioned vs unconditioned MEP amplitude for the first TMS block in each session was significantly less than 1; 1 ms SICI: *t*(12) = 5.92, *P* < 0.001; 2.5 ms SICI: *t*(12) = 3.47, *P* = 0.05). We therefore present all ppTMS data in terms of the change over the intervention to simplify our results. The mean, standard deviation, maximum and minimum inhibition induced for each of the ppTMS protocols is given in Table 1.

1 ms SICI and 2.5 ms SICI have previously been shown to be mechanistically independent [30], [31], and therefore the effects of stimulation and task performance were analyzed for each SICI type separately.

#### Task performance modulates extrasynaptic GABA tone (as assessed by 1 ms SICI)

We first investigated the effects of stimulation on 1 ms SICI. Neither tDCS alone (**A**-0) nor tDCS delivered concurrently with task performance (**AT**-0) modulated 1 ms SICI (RM-ANOVA, main effect of time: *F*(1,12) = 0.487, *P* = 0.499).

We next considered effects of task on 1 ms SICI. Task performance significantly increased 1 ms SICI across the three task sessions (**AT**-0, A-**T** and S-**ST**; RM-ANOVA, main effect of time: *F*(1,12) = 6.073, *P* = 0.030), but this change in 1 ms SICI did not differ between sessions (time × session interaction: (*F*2,24) = 1.10, *P* = 0.346). In order to ensure that changes in 1 ms SICI were not solely due to the passage of time, we tested for a change in 1 ms SICI during the sham-only time period (i.e. the first portion of the **S**-ST session), and found no change in 1 ms SICI (*t*(12) = 0.299, *P* = 0.770).

#### Task performance modulates GABA_A_ synaptic activity

An identical series of analyses to those for the 1 ms SICI data was then performed for 2.5 ms SICI in order to test for effects of stimulation or task performance on GABA_A_ synaptic activity. Contrary to previous findings [22], neither tDCS alone (**A**-0) nor tDCS delivered concurrently with task performance (**AT**-0) modulated 2.5 ms SICI (RM-ANOVA, main effect of stimulation: *F*(1,12) = 1.39, *P* = 0.26). Previous studies have demonstrated a significant decrease in MRS-assessed GABA levels with anodal tDCS [21], [28]. It is likely that this measure most closely reflects change in both 1 ms SICI and 2.5 ms SICI. Therefore we tested the effects of anodal tDCS on the average of these two measures. There was a trend towards a significant decrease in this combined measure of inhibition after anodal tDCS when applied alone, but not when applied with a task (**A**-0: *t*(12) = 1.51, *P* = 0.07; **AT**-0: *t*(12) = -0.44, *P* = 0.70; data not shown).

**Figure 4:**
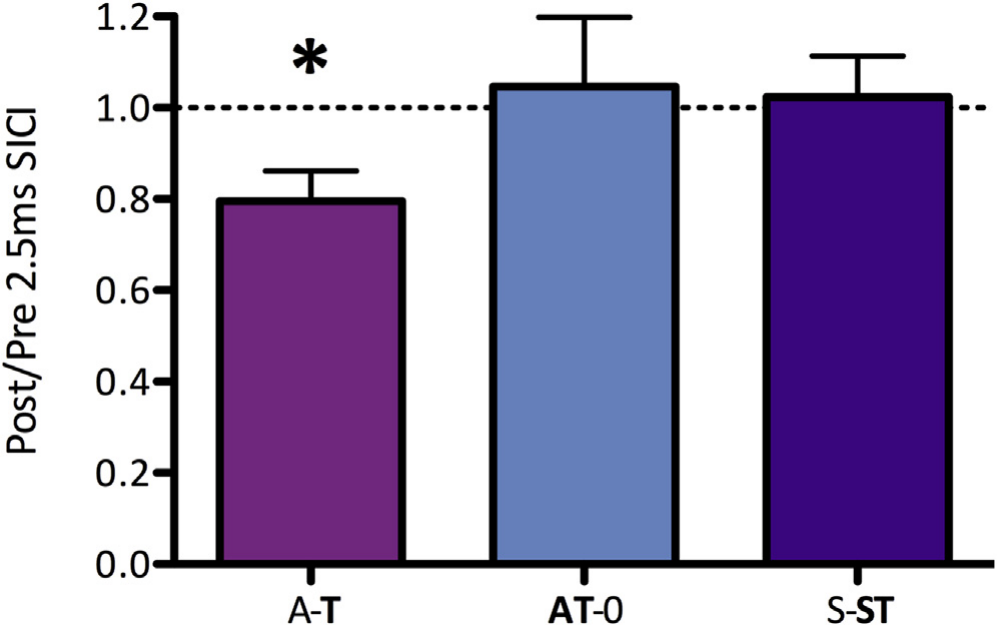
Task performance increases GABAA-synaptic inhibition. Changes in inhibition expressed as a ratio of post- to pre- task performance 2.5ms SICI. Anodal tDCS applied prior to task performance (A-T), increases task-related GABAA activity as measured by 2.5ms SICI. Note that lower values reflect greater inhibition. Error bars ±1 SEM, * *P* < 0.05.

We next tested the effects of task performance on 2.5 ms SICI. Task performance modulated GABA_A_ activity (RM-ANOVA, main effect of time: *F*(1,12) = 8.132, *P* = 0.015; Fig. 4 [note that smaller values reflect greater inhibition]). Post-hoc *t*-tests demonstrated a significant *increase* in 2.5 ms SICI after task performance when the task was performed after tDCS (A-**T**: t(12) = 2.983, *P* = 0.011), but no modulation of 2.5 ms SICI due to task performance in the absence of stimulation (S-**ST**: t(12) = 0.502, *P* = 0.625), nor when the task was performed during stimulation (**AT**-0: t(12) = 0.785, *P* = 0.448).

#### Changes in GABA_A_ synaptic activity after anodal tDCS are behaviorally relevant

**Figure 5:**
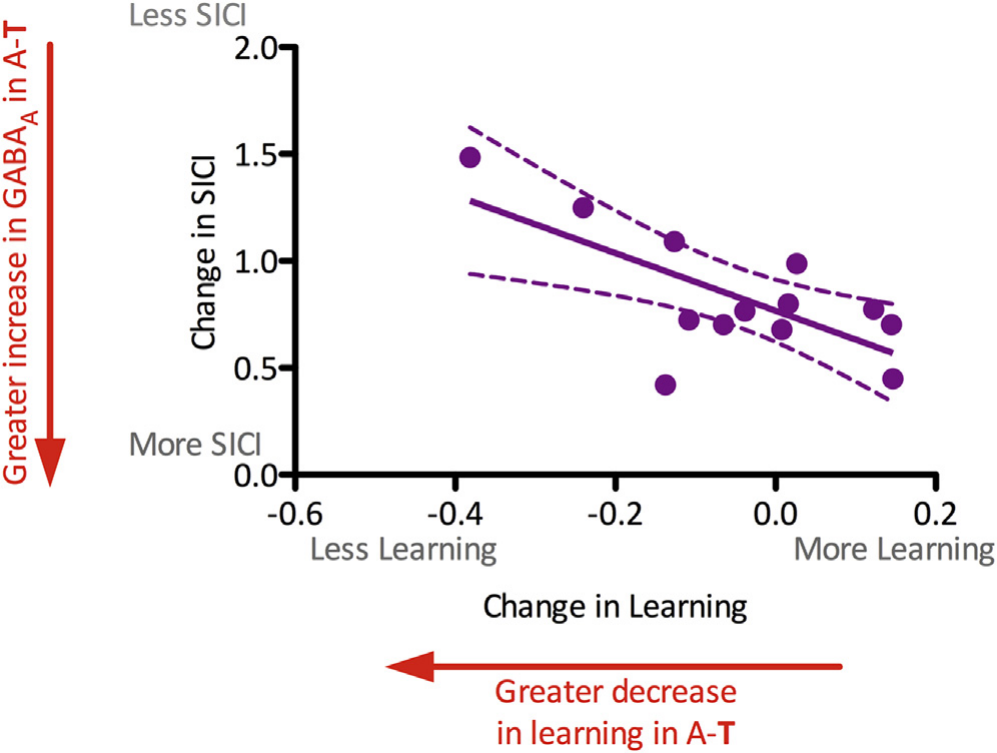
Worsening of learning after anodal tDCS is inversely related to increase in GABA_A_ activity in the same period. The decrease in learning of the motor task when task performance was preceded by anodal tDCS (A-**T**) is correlated with the increase in 2.5 ms SICI seen over the same period (*r* = −0.69, *P* = 0.009), such that subjects who showed a less detrimental behavioral effect of prior anodal tDCS (i.e. those who were presumably able to induce more LTP-like plasticity during the task) were those in whom the increase in GABA_A_ activity was greatest over the same time period.

We then went on to investigate the behavioral relevance of the increase in 2.5 ms SICI seen after task performance when the task was performed after anodal tDCS (i.e. A-**T**). We correlated this increase in 2.5 ms SICI seen after learning in the A-**T** session (Fig. 4) with the degree of the decrease in learning over the same period (Fig. 2). The decrease of learning in the A-**T** session was calculated as the decrease in RTs (ΔRT) when the task was performed after anodal stimulation (i.e. A-**T**) compared with the ΔRT when the task was performed after sham stimulation (i.e. S-**ST**). There was a highly significant relationship between the decrease in learning and the decrease in GABA over this time period, such that people who showed less of a decrease in learning rates when performing the task after anodal tDCS were also those who showed a greater increase in SICI over the same time period (*r* = −0.69, *P* = 0.009; Fig. 5).

This relationship was specifically found for the A-**T** session. There was no relationship between learning rates *per se* (i.e. when learning occurred in the S-**ST** session) and the change in 2.5 ms SICI occurring during that learning (S-**ST**; *r* = 0.31, *P* = 0.3). Indeed, the relationship between the decrease in learning and the increase in SICI observed during learning when performed after prior anodal tDCS (A-**T**) was significantly stronger the relationship between learning and SICI after sham stimulation (i.e. S-**ST**; Fisher's *r* to *Z*; *Z* = 2.61, *P* = 0.01).

We went on to investigate whether the decrease in learning in the A-**T** session could be explained by changes in 2.5 ms SICI induced by the preceding period of anodal stimulation (i.e. **A**-T), even if no overall change in this metric was seen. We found a significant relationship between the decrease in learning in the A-**T** session and the change in 2.5 ms SICI in the **A**-T session, such that subjects who showed a greater decrease in 2.5 ms SICI in the first period of the session were those who showed greater decrease in subsequent motor learning (*r* = 0.50, *P* = 0.035).

We then wished to investigate the specificity of the relationship between change in motor learning and change in GABA_A_ activity in the A-**T** session. One way to do this might be to investigate whether there was a relationship between the worsening of learning in the A-**T** session and the change in overall cortical excitability, as assessed by MEP change, over the same period. Change in MEPs in the A-**T** session was calculated as the change in MEPs when the task was performed after anodal stimulation (i.e. A-**T**) compared with the change in MEPs when the task was performed after sham stimulation (i.e. S-**ST**). There was no significant relationship between the worsening of learning after anodal tDCS (i.e. in the A-**T** session) and the change in overall cortical excitability as assessed by change in MEPs (*r*(12) = -0.08, *P* = 0.786). Neither was there any significant relationship between the change in MEPs, when calculated from the A-**T** condition alone and the worsening of learning over the same period (*r*(12) = 0.005, *P* = 0.98). These findings suggest that the observed relationship between change in 2.5 ms SICI and worsening of learning over the same period was specific to GABA_A_ synaptic activity.

## Discussion

This study was performed to investigate the previously described homeostatic interactions between anodal tDCS and motor learning. Specifically, we wished to investigate the role of GABA_A_ synapses, which are causally modulated by both interventions, as a putative site for this interaction to occur. To this end we used TMS to measure cortical excitability, GABA_A_ synaptic activity, and GABAergic tonic inhibition.

We first confirmed that the relative timing of anodal tDCS and motor learning is critical to the behavioral effects of tDCS on learning: learning was slowed by prior anodal tDCS (A-**T**) but not by concurrent tDCS (**AT**-0; Fig. 2). We then showed that the decrease in learning rates induced by preceding anodal tDCS (A-**T**) was related to increased synaptic GABA_A_ activity over this same time period, as assessed by 2.5 ms SICI (Figure 4, Figure 5), suggesting that inhibitory processes play a role in this homeostatic relationship. The hypothesis that the relationship between anodal tDCS and motor learning has a synaptic basis was further strengthened by the lack of an effect of the interaction of tDCS and motor learning on 1 ms SICI, a putative measure of extrasynaptic GABAergic tone.

We were particularly interested in the role of GABA_A_ synapses, as they have been strongly implicated in plasticity in the motor cortex [21], [22], [23], [24]. Plasticity within the primary motor cortex is driven, at least in part, by rapid remapping of cortical representations. There is extensive evidence to suggest that this remodeling is related to the pre-existing architecture of GABAergic horizontal connections [32], [33], and these connections can be unmasked by the blockade of GABA_A_ receptors [34]. Further, a reduction in GABA_A_ activity facilitates Long Term Potentiation (LTP)-like plasticity in M1 [35], [36]. In humans, pharamacologically increasing GABA_A_ synaptic activity leads to a worsening of motor learning [37], [38]. For a full review of the role of GABA in human motor plasticity see Ref. [39].

### tDCS and motor learning interact in a homeostatic manner

Applying anodal tDCS prior to task performance (A-**T**) resulted in decreased learning compared to the control session (S-S**T**; Fig. 2), consistent with homeostatic mechanisms. Homeostatic rules state that increased background activity leading to LTP-like synaptic changes prevents further facilitation by subsequent excitatory stimuli. Anodal tDCS increases background activity by increasing neuronal firing rates [40], an effect demonstrated in TMS measures as an increase in MEP size [1]. This increased background activity has been shown to induce LTP-like plasticity [41]. In this heightened state, then, processes, such as motor learning, which rely on inducing on LTP-like changes should be blocked, as seen here.

### Homeostatic interactions may occur at GABA_A_ synapses

Although the finding of a homeostatic interaction between anodal tDCS and motor learning is suggestive that the two interventions modulate a similar set of microcircuits within the motor cortex, it cannot inform us as to which synapses are involved in this process.

Previous studies have suggested that both tDCS and motor learning modulate local inhibitory processing [21], [22], [23], [24] and GABA modulation by tDCS has been shown to be related on a subject-by-subject basis to the degree of learning of a motor task performed on a different day [28]. A previous study has suggested that homeostatic interactions between trains of theta burst TMS are associated with modulation of GABA_A_ synapses [18]. We therefore wanted to investigate whether homeostatic interactions between anodal tDCS and motor learning were driven, at least in part, by GABA_A_ synaptic activity.

Here, we had two distinct measures of local inhibitory activity. 2.5 ms SICI is a relatively specific measure of GABA_A_ synaptic activity [26], [42], [43]. Less is understood about 1 ms SICI, though it is known to be GABA-dependent [44], distinct from 2.5 ms SICI [30], [31] and has been previously suggested to reflect extrasynaptic GABA tone [25].

Our results suggest that extrasynaptic GABA tone and GABA_A_ synaptic activity are differentially modulated by stimulation and learning. Consistent with the idea that homeostatic plasticity is a synaptic phenomenon, 1 ms SICI was increased after learning, but this effect was not modulated by the relative timing of tDCS and learning. The effects of learning on 2.5 ms SICI, however, *were* dependent on the relative timing of the stimulation: GABA_A_ synaptic activity was significantly increased after learning when the learning task was preceded by tDCS (A-**T**), but no measurable change occurred in the absence of stimulation (S-S**T**) or when stimulation was applied during the learning task (A**T**-0; Fig. 4).

That more inhibition is associated with decreased learning conforms to the theory of homeostatic plasticity. Referring to the “plasticity of synaptic plasticity” [45], homeostatic plasticity describes synaptic changes that regulate the ability for LTD or LTP to be induced over time. One particularly influential model of homeostatic plasticity is the Bienenstock-Cooper-Munro (BCM) theory. The BCM model proposes that there is a level of post-synaptic activity, termed the modification threshold (*θ*_m_), above which LTP results and below which LTD results [15]. The modification threshold is a dynamic entity, and can be raised or lowered based on the previous time-averaged level of post-synaptic cell firing. Accordingly, anodal tDCS, which increases post-synaptic activity, would raise the *θ*_m_, decreasing the likelihood of LTP formation and increasing the likelihood of LTD. Thus the level of task-induced post-synaptic activity that would normally result in LTP may now induce LTD and, as a result, decreased learning and greater cortical inhibition.

According to the BCM model, homeostatic plasticity occurs via modifications in synaptic mechanisms and is well characterized for glutamatergic changes [46], [47]. It is important to note that 2.5 ms SICI reflects activity within GABAergic microcircuits within the cortex. The exact nature of these has yet to be fully characterized, but they almost certainly contain glutamatergic synapses in addition to GABA_A_ synapses. We cannot be certain, therefore, that any homeostatic interactions are occurring at the level of the GABA_A_ synapse, but rather can only conclude that they are occurring within the GABAergic cortical microcircuits stimulated by the SICI protocol.

The contribution of GABA to such homeostatic processes has long focused on its ability to indirectly shift *θ*_m_ via increasing the NMDA response and intracellular Ca^2+^ concentration [48], or by auto-regulatory inhibition of GABA release as a result of post-synaptic excitation of neighboring terminals [49]. However, recent work suggests that specific inhibitory interneuronal subtypes show homeostatic plasticity in the spinal dorsal horn [50]; the hippocampus [51], [52]; and the neocortex [18], [53], suggesting these as a potential site for the homeostatic interactions seen here.

#### Why does 2.5 ms SICI *increase* after motor learning following tDCS?

Neither anodal stimulation alone (**A**-0), motor learning alone (S-**ST**) nor anodal tDCS applied concurrently with motor learning (**AT**-0) led to a change in GABA_A_ activity. However, when motor learning was performed *after* anodal tDCS (A-**T**, the session in which impaired motor learning was observed) a significant concurrent increase in 2.5 ms SICI was seen, suggesting an increase in GABA_A_ activity. Further, subjects who showed a behaviorally less detrimental effect of prior anodal tDCS (i.e. those who showed greater learning (more positive values on *x*-axis, Fig. 5) and so were presumably able to induce more LTP-like plasticity during the task) were those in whom the increase in 2.5 ms SICI (reflecting greater GABA_A_ activity) was greatest (smaller values on *y*-axis, Fig. 5). The direction of this relationship supports the notion that the amount of LTP-like plasticity induced during learning after anodal tDCS was critical in determining the degree of GABA_A_ homeostatic response to that learning.

This would be consistent with the BCM model of homeostatic plasticity, which states that an intervention (in this case motor learning) which would normally be expected to induce LTP-like changes will induce LTD-like changes if performed after another intervention (here tDCS) which also modulates synaptic plasticity.

Does this interaction give us any information as to the likely common site of action for anodal tDCS and motor learning? Homeostatic interactions are seen in many contexts in the brain and, as well as mechanisms subserving slow, global homeostatic corrections, rapid, synapse- or micro-circuit- specific interactions are increasingly being recognized, in particular those occurring in specific populations of GABA_A_ interneurons [47]. A homeostatic interaction between two interventions on this timescale therefore suggests that similar populations of inhibitory interneurons are stimulated by both interventions, further suggesting these interneurons as a putative substrate for the positive behavioral effects of tDCS on motor learning that have been previously described.

### Why does on-line tDCS not affect motor learning?

Our finding that anodal tDCS applied concurrently with the motor task does not modulate motor learning (A**T**-0) is at odds with previous literature suggesting a speeding of learning with concurrent tDCS [6], [7], [8]. There are a number of possible reasons for this. It might be that the explicit sequence learning task performed here is less sensitive to modulation by tDCS, but learning a very similar task has previously been shown to be improved by concurrent stimulation [6]. However, there is a major difference between the task used here and that used in our previous 2011 paper. Here, the 30 s task blocks were separated by 30 s rest periods, much longer than those we, and others, have used previously [6], [7]. Given the slowing of learning seen when the task was performed after stimulation (A-**T**), it is possible that the anodal tDCS-induced increase of learning during task performance was counterbalanced by a tDCS-induced slowing of learning due to the tDCS administered while no learning was happening in the rest blocks. While this is a parsimonious explanation of the data in light of the preceding literature, we do not have sufficient data to confirm or refute it here.

Previous studies have shown an increase in MEP amplitude following a period of motor learning [13], [24]. We do not see such an increase here. Although we cannot be certain of why we do not replicate this increase, one explanation may be that previous studies have employed a learning task whereby subjects were required to abduct their thumb as quickly as possible. Here, rather than focusing learning on one muscle we used a task that involved many muscles within the hand. It may be that cortical excitability is less significantly modulated by this more generalized learning task than one involving a single muscle, though this hypothesis needs further investigation.

## Conclusions

In this study we aimed to investigate the physiological mechanisms subserving the previously described homeostatic relationship between anodal tDCS and motor learning. We have shown that when motor learning is preceded by anodal tDCS, learning rates are slowed and GABA_A_ activity increased. These results are the first to our knowledge to explore the physiological underpinnings of homeostatic interactions between stimulation and learning in humans. They are important as they support a potential role for GABA_A_ synapses in human motor homeostatic plasticity, and further add weight to the hypothesis that anodal tDCS may modulate behavior at least in part through modulation of GABA_A_ synapses.

## Figures and Tables

**Supplementary Figure 1:**
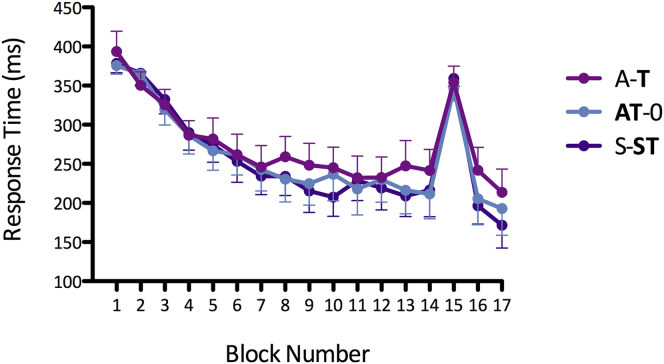


**Table 1:** Mean, standard deviation, minimum and maximum inhibition induced by each of the ppTMS protocols.

ppTMS measure	Inhibition			
Mean	S.D.	Min	Max
1 ms SICI	0.56	0.08	0.65 (A-0, final block)	0.43 (A-T, final block)
2.5 ms SICI	0.79	0.07	0.90 (A-0, mid block)	0.67 (A-T, final block)
