# Supplementary material for: The Homeostatic Interaction Between Anodal Transcranial Direct Current Stimulation and Motor Learning in Humans is Related to GABAA Activity
Source: Brain Stimul. 2015 Sep-Oct;8(5):898–905. doi: 10.1016/j.brs.2015.04.010 (PMC4742653; doi:10.1016/j.brs.2015.04.010)
Supplement: Supplementary Table 1 [file mmc1.docx]

MEP amplitude

| Session | Pre | Mid | Post |
| --- | --- | --- | --- |
| A-0 | 0.90 ± 0.13 | 1.167 ± 0.18 | 1.11 ± 0.17 |
| A-T | 1.14±0.10 | 1.20±0.11 | 1.18±0.13 |
| AT-0 | 1.56±0.12 | 1.41±0.18 | 1.55±0.22 |
| S-ST | 1.10±0.14 | 1.50±0.21 | 1.21±0.15 |

MT_1mV_

| Session | Pre | Mid | Post |
| --- | --- | --- | --- |
| A-0 | 62.3±2.17 | 60.7±2.51 | 61.1±2.52 |
| A-T | 62.3±2.42 | 62.1±2.36 | 62.7±2.49 |
| AT-0 | 61.6±2.29 | 61.3±2.41 | 61.0±2.38 |
| S-ST | 61.2±2.12 | 61.8±2.11 | 62.0±2.20 |

AMT

| Session | Pre | Mid | Post |
| --- | --- | --- | --- |
| A-0 | 26.0±0.86 | 26.0±0.86 | 26.0±0.86 |
| A-T | 26.6±0.88 | 26.6±0.88 | 26.6±0.88 |
| AT-0 | 25.9±0.88 | 25.9±0.88 | 25.9±0.88 |
| S-ST | 26.3±0.91 | 26.3±0.91 | 26.3±0.91 |

## Table Legend

Mean ± SE values for (A) MEP amplitude (B) MT_1mV_ and (C) AMT for each TMS block across the four experimental sessions.
