# Supplementary material for: The Homeostatic Interaction Between Anodal Transcranial Direct Current Stimulation and Motor Learning in Humans is Related to GABAA Activity
Source: Brain Stimul. 2015 Sep-Oct;8(5):898–905. doi: 10.1016/j.brs.2015.04.010 (PMC4742653; doi:10.1016/j.brs.2015.04.010)
Supplement: Legend for Supplementary Figure 1 [file mmc2.docx]

# Figure Legends

## Supplementary Figure 1

Reaction times for each block of the motor task, plotted as mean ± SEM.
